# Impact of a Soil Conditioner Integrated into Fertilization Scheme on Orange and Lemon Seedling Physiological Performances

**DOI:** 10.3390/plants9070812

**Published:** 2020-06-28

**Authors:** Lorenzo Rossi, Lukas M. Hallman, Sawyer N. Adams, Walter O. Ac-Pangan

**Affiliations:** Horticultural Sciences Department, University of Florida, Institute of Food and Agricultural Sciences, Indian River Research and Education Center, Fort Pierce, FL 34945, USA; lukas.hallman@ufl.edu (L.M.H.); sawyer.adams@ufl.edu (S.N.A.); acpangan@live.com (W.O.A.-P.)

**Keywords:** citrus physiology, fertilization, soil amendment

## Abstract

Growers in Florida face unique challenges regarding maintaining proper citrus nutrition. Poor draining soils with low fertility, low C.E.C., and high rates of leaching are common in this region. In response to these challenges, interest has grown in products labeled as soil conditioners. Using a completely randomized experimental design, this greenhouse study tested the effects of 5 different combinations of a traditional fertilizer (TF) and a new soil conditioner (SC) on lemon and orange seedling physiology. Eight-month-old ‘Bearss’ lemon and ‘Valencia’ sweet orange grafted on sour orange rootstocks were employed, and five repetitions were used for each treatment. Plant biomass (dry weight), height, stem diameter, chlorophyll content, stomatal conductance and nutrient uptake were analyzed after 120 days of treatment. The results show that SC has a positive impact upon both chlorophyll levels and stomatal conductance values in both orange and lemon seedlings. However, based on dry weight growth data, we can only conclude that the SC was effective for orange seedlings at 50% TF and 0.5% SC. Based on this short 120-day evaluation, the SC achieved positive growth promotion for orange (50% TF) but not for lemon seedlings.

## 1. Introduction

Citrus production is a multi-billion-dollar industry with growing regions in over 130 countries worldwide. Brazil, China, the United States, Mexico, and the Mediterranean basin produced over 80% of the estimated 124,000,000 tons of citrus grown in the world in 2016 [1]. Due to the subtropical climate required for growing citrus [2], commercial citrus production in the United States is limited to California, Florida, Texas, and Arizona. Total production in 2018 was valued at just over three billion dollars [3].

Mineral nutrition plays a vital role in sustaining the production of the global citrus industry. Proper nutrient supply positively impacts plant growth, fruit production, anatomy, and morphology [4,5]. Among the many plant essential nutrients, nitrogen (N), phosphorus (P), and potassium (K) are required in large quantities and are required for several processes [6]. For example, N affects photosynthetic rates, carbohydrate synthesis, and biomass production [7,8,9]. Additionally, P is an important component of plant growth and development due to its structural role in DNA, RNA, and ATP [10]. Furthermore, K is linked to carbohydrate transport, cell elongation, opening and closing of stomata, and enzyme activation [5].

The availability of mineral nutrients to plants depends largely on soil characteristics [11]. The soils of Florida’s different growing regions pose a challenge for maintaining adequate plant nutrition [12,13,14,15], particularly the Indian River growing region, which is located on the central east coast of Florida [13,15]. The soils in this region are vastly different from the soils in Florida’s central ridge growing region and thus require different management strategies [16]. These soils are appropriately named flatwood soils, and are characterized as being sandy with a slowly permeable subsurface layer [17]. Additionally, the soils have low fertility, low C.E.C., poor drainage, and high pH [18]. Due to these soil characteristics, the Indian River region is prone to leaching of applied fertilizers, and thus subject to reduced nutrient availability [6]. In addition to challenging soil characteristics, growers in the Indian River region must also consider the impacts of different diseases on nutrient management, particularly the bacterial disease Huanglongbing (HLB; also known as citrus greening) which damages root systems and thereby reduces nutrient uptake in affected plants [19].

In response to the challenges of HLB, improved nutrient guidelines are being developed to prolong the productive lifespan of citrus and make Florida’s citrus production more profitable [20,21]. In addition to studies on increased nutrient concentrations, some research has been conducted on products labeled as soil conditioners and root growth enhancers. By definition, soil conditioners are amendments that when added to soil, may improve physical qualities and processes. A study conducted by Xu et al. [22] demonstrated that citrus treated with soil conditioner had increased fruit yield and quality, increased soil organic matter, and elevated soil nutrient levels of nitrogen (N), phosphorus (P), potassium (K), and manganese (Mn) in comparison to non-treated citrus. In response to such results, some companies have begun marketing and selling root growth enhancers and conditioners [23,24]. In many instances, these products are marketed to growers with little scientific evidence regarding their effectiveness. Therefore, it is important to conduct both greenhouse and field trials to determine if these products are effective at increasing root mass, and if so, how these products cause additional root mass to occur. 

One such product, oGrowing™, is a liquid root growth enhancer made of dormant oceanic diatoms. The manufacturer asserts that the application of these diatoms to the soil enhances the bioavailability of nutrients in the root zone, resulting in increased efficiency in plant nutrient uptake [25]. This product is currently being used in some commercial groves and has garnered interest from Florida growers in the Indian River region. 

This study evaluated the physiological responses of orange and lemon seedlings subjected to varying traditional fertilizer concentrations (TF; i.e., Osmocote® Plus) in conjunction with a soil conditioner (SC; i.e., oGrowing™). The impact of the soil conditioner on selected physiological indices was also observed. Considering the challenges of maintaining proper nutrition in Florida’s citrus growing regions [26], it is important to investigate the physiological response citrus has to varying levels of the selected fertilizer and root growth enhancers, so that improved nutrient management strategies can be devised.

## 2. Results

### 2.1. Plant Biomass (Dry Weight)

**Figure 1:**
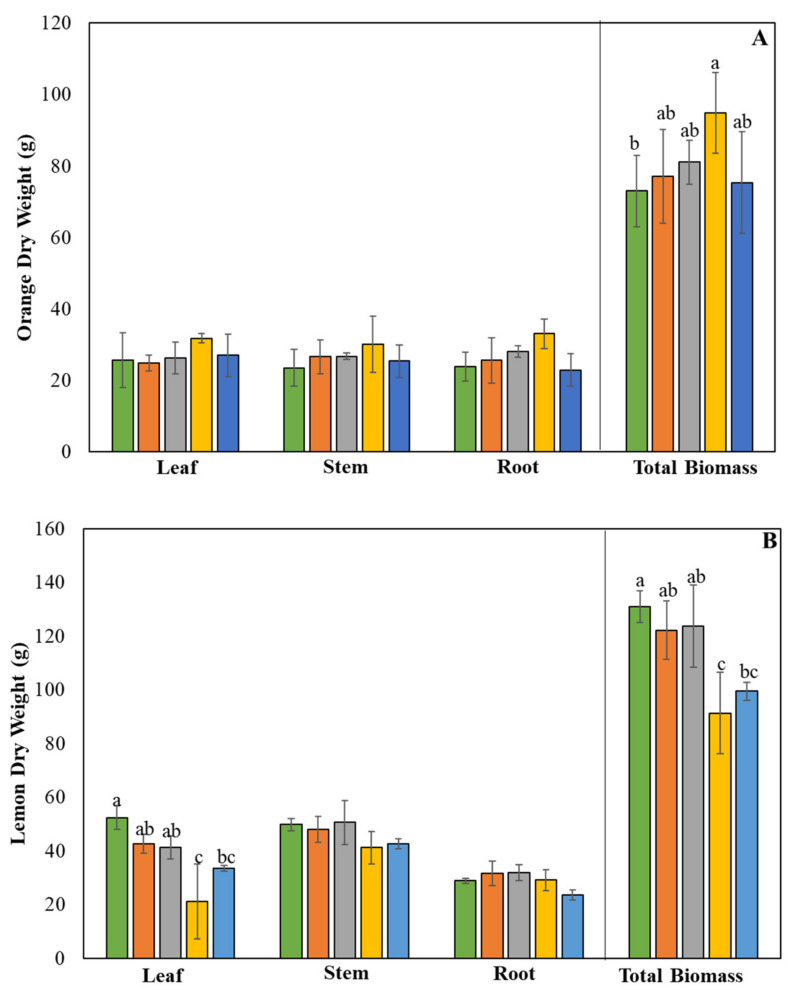
Dry weight of orange (**A**) and lemon (**B**) seedlings grown in the presence of 100% or 50% TF (traditional fertilizer) and/or 0.5 or 1% SC (soil conditioner). Means labeled by different letters are significantly different by Tukey’s post-hoc test (*p* < 0.05). Error bars represent the standard deviation (*n* = 5).

No significant differences were detected in the dry weight (DW) of orange seedling leaf, root, or stem but total biomass showed significant difference when seedlings were treated with 50% TF and 0.5% SC (Figure 1A). In particular, only the treatment with 50% TF and 0.5% SC attained the best growth. Unlike the orange seedlings, a significant difference was observed in the leaf DW and total biomass of the lemon seedlings, but the usefulness of the SC for lemon was not demonstrated (Figure 1B). At 100% TF, adding SC did not show significant changes. At 50% TF, adding SC reduced the total biomass of the saplings.

### 2.2. Plant Growth and Development (Height and Stem Diameter)

**Figure 2:**
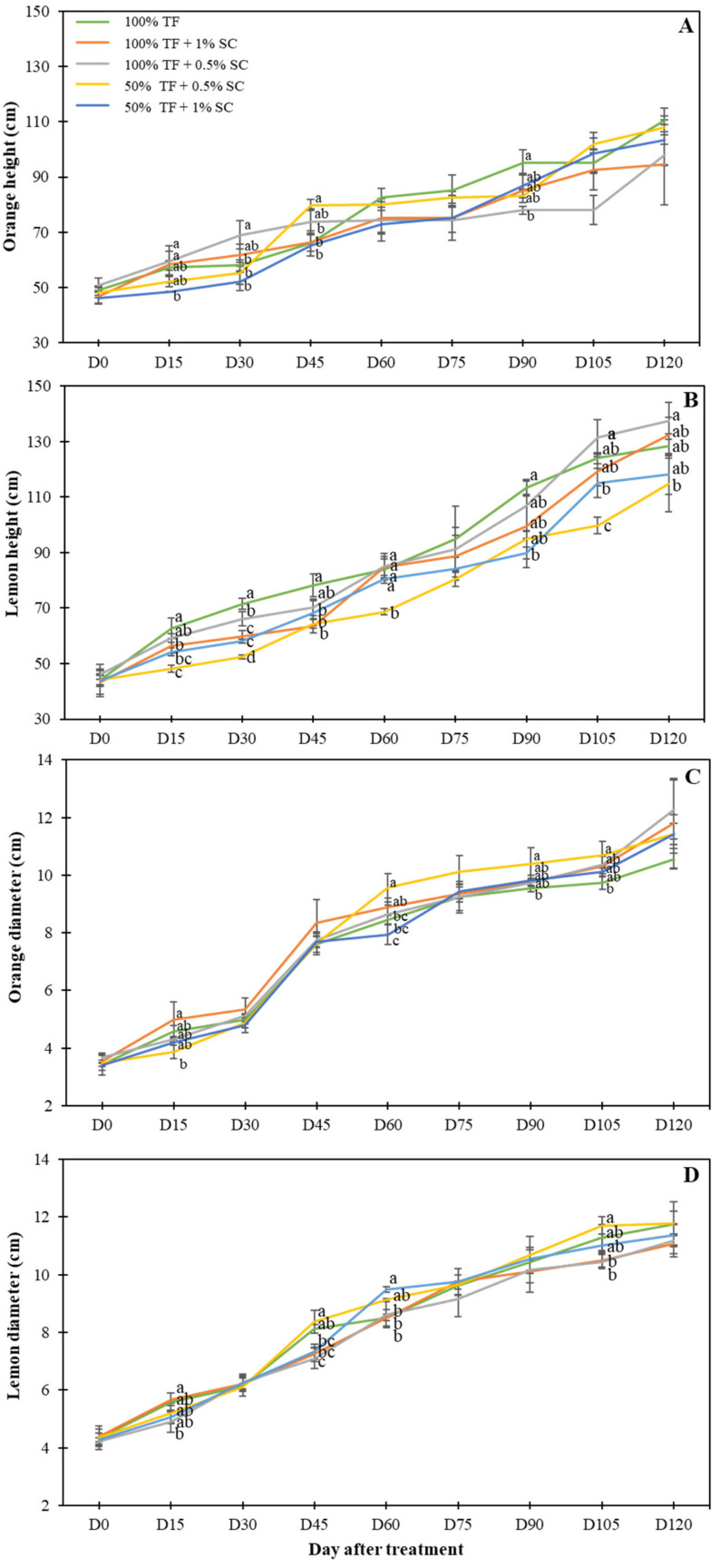
Height (**A**,**B**) and diameter (**C**,**D**) of orange (**A**,**C**) and lemon (**B**,**D**) seedlings grew in the presence of 100% or 50% TF fertilizer and/or 0.5 or 1% SC for 120 days (D120). Means labeled by different letters are significantly different by Tukey’s post-hoc test (*p* < 0.05). Error bars represent the standard deviation (*n* = 5).

Significant differences in plant height (cm) of orange seedlings were recorded at day 15 (D15), day 30 (D30), day 45 (D45), and day (D90). At D90, control seedlings had an average height increase of 64.26% compared to seedlings treated with 100% TF + 0.5% SC which only had a height increase of 42.24% (Figure 2A). Additionally, at D90, control seedlings were 19.86% taller compared to seedlings treated with 100% TF + 0.5% SC. Dissimilarly, the lemon seedlings had significant height changes beginning at D15 and continuing until D120, excluding D75. Seedlings treated with 100% TF + 0.5% SC had an average height increase of 99.23% compared to seedlings treated with 50% TF + 0.5% SC, whose average height increased 88.99% (Figure 2B). Additionally, seedlings treated with 100% TF + 0.5% SC were 17.99% taller compared to seedlings treated with 50% TF + 0.5% SC (Figure 2B). 

Significant differences in orange seedling stem diameter were observed at D15, D60, D90, and D105. At D105, seedlings treated with 50% TF + 0.5% SC had a 97.22% increase in diameter, while control seedlings only had a 96.18% increase (Figure 2C). The diameter of orange seedlings treated with 50% + 0.5% SC was 9.80% larger than seedlings treated with the control treatment. Similar observations were recorded in lemon seedlings. Significant differences between treatments were observed at D15, D45, D60, and D105. At D105, seedlings treated with 50% TF + 0.5% SC had significantly larger diameters compared to 100% TF + 1% SC and 100% TF + 0.5% SC-treated seedlings (10.81% larger) (Figure 2D). 

### 2.3. Chlorophyll Contents

**Figure 3:**
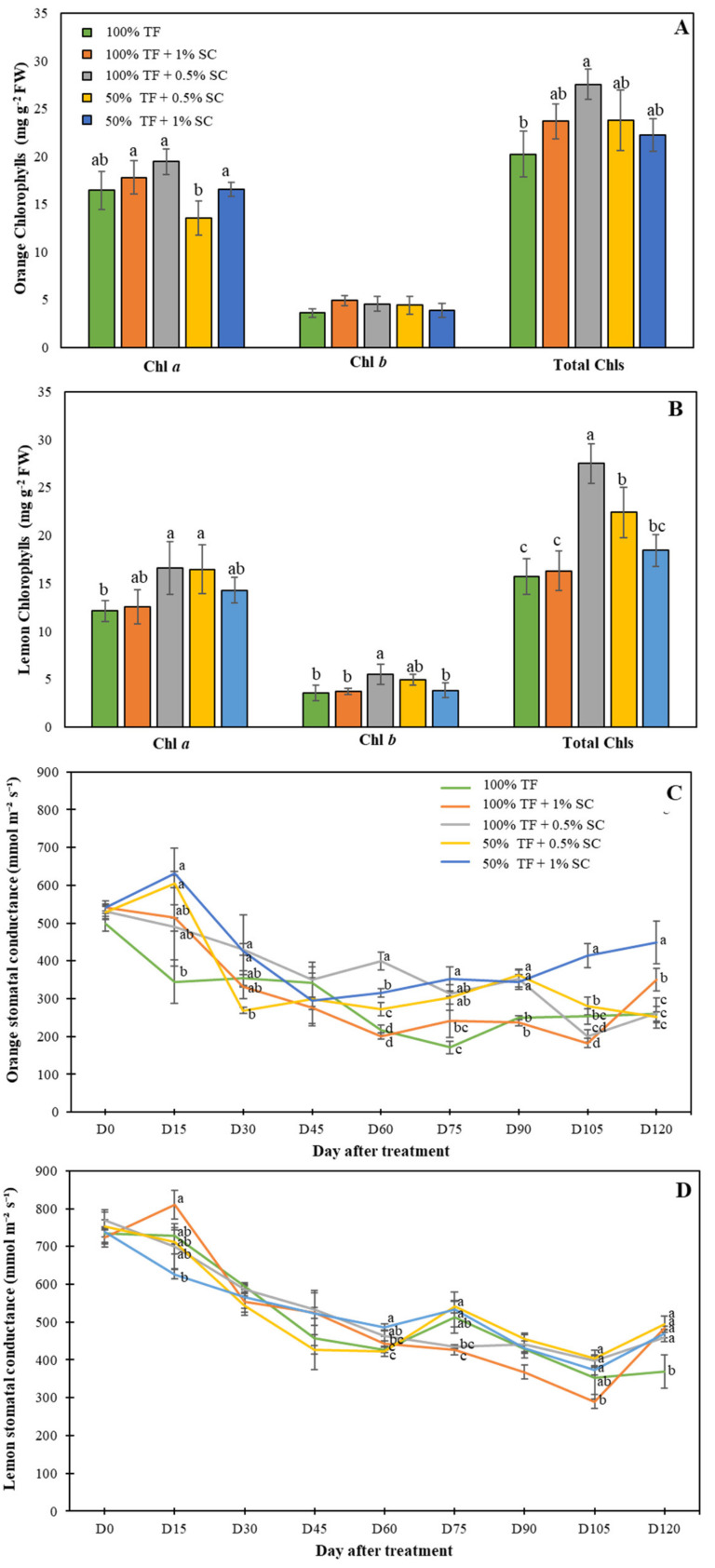
Chlorophyll contents (**A**,**B**) and stomatal conductance (**C**,**D**) of orange (**A**,**C**) and lemon (**B**,**D**) seedlings grew in the presence of 100% or 50% TF fertilizer and/or 0.5 or 1% SC for 120 days (D120). Means labeled by different letters are significantly different by Tukey’s post-hoc test (*p* < 0.05). Error bars represent the standard deviation (*n* = 5).

In orange seedlings, no significant differences were detected in chlorophyll *b* content. However, chlorophyll *a* content was significantly higher (44.71%) in seedlings treated with 100% TF + 1% SC and 100% TF + 0.5% SC compared to the control group. This was also reflected in the total chlorophyll content (Figure 3A). Significant differences in both chlorophyll *a* and *b* content were recorded in lemon seedlings. Seedlings treated with 100% TF + 0.5% SC had 45.03% higher chlorophyll *a* than control seedlings (Figure 3B). Additionally, seedlings treated with 100% TF + 0.5% SC had higher chlorophyll *b* content than control (50 % higher), 50% TF + 0.5% SC (15.69% higher), and 50% TF + 1% SC (58.82% higher) treatment groups. Total chlorophyll content confirmed this trend (Figure 3B).

### 2.4. Stomatal Conductance

Significant differences in the stomatal conductance of orange seedlings were seen on D15 through D120, except D45. At the end of the experiment, orange seedlings treated with 50% TF + 1% SC had 24.39% higher rates of stomatal conductance than seedlings treated with 100% TF + 1% SC, and averaged 54.12% higher than the control, 100% TF + 0.5% SC, and 50% TF + 0.5% SC treatment groups (Figure 3C). However, a different trend was observed in lemon seedlings (Figure 3C). Significant differences in stomatal conductance were first recorded at D15 and continued until D120 except on D30, D45, and D90. At D120, all treatments had significantly higher stomatal conductance levels (average of 25.59%) compared to the control treatment (Figure 3D).

### 2.5. Nutrient Concentrations

**Figure 4:**
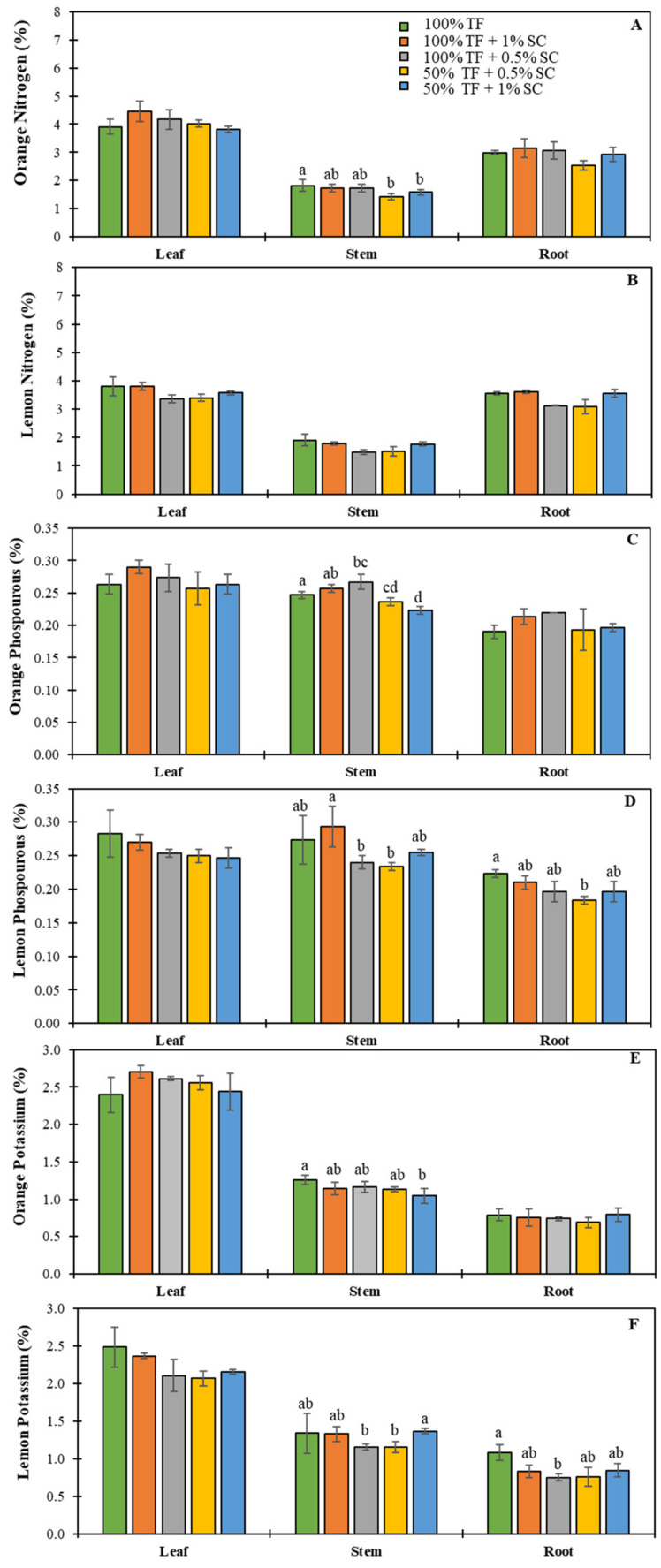
Nutrients (N, P and K) contents in orange (**A**,**C**,**E**) and lemon (**B**,**D**,**F**) seedlings grew in the presence of 100% or 50% TF fertilizer and/or 0.5 or 1% SC for 120 days (D120). Means labeled by different letters are significantly different by Tukey’s post-hoc test (*p* < 0.05). Error bars represent the standard deviation (*n* = 5).

A significant difference was observed in N concentration in the stems of orange seedlings. Seedlings given the control treatment had N levels 22.22% higher than seedlings given the 50% TF + 0.5% SC treatment and 11.11% higher than seedlings given the 50% TF + 1% SC treatments (Figure 4A). Significant differences in P concentrations were detected in the stems of orange seedlings. The control treatment had 7.81% less P compared to the 100% TF + 0.5% SC and 9.81% more compared to 50% TF + 1% SC (Figure 4C). Remarkably, significant differences in P were observed in both lemon stems and lemon roots. Lemon seedlings treated with 100% TF + 1% SC had higher concentrations of P in the stems compared to seedlings treated with 100% TF + 0.5% SC (18.87 % higher) and 50% TF + 0.5% SC (23.08% higher) (Figure 4D). In lemon roots, control seedlings had 19.26% higher levels of P compared to seedlings treated with 50% TF + 0.5% SC. Oranges seedlings treated with the control treatment had 19.13% higher concentration of K compared to seedlings treated with the 50% TF + 1% SC treatment (Figure 4E). No significant differences were observed in K levels in the leaves or roots of orange seedlings. Significant differences were recorded in the K levels of both stem and roots of lemon seedlings. In the stems, lemon seedlings treated with 50% TF + 1% SC had 15.29% more K than seedlings treated with 100% TF + 0.5% SC and 16.18% more K then seedlings treated with 50% TF + 0.5% SC (Figure 4F). The root K levels of the control seedlings were 36.07 % higher than seedlings treated with 100% TF + 0.5% SC (Figure 4F). 

## 3. Discussion

This study investigated the impact of different concentrations of fertilizer in combination with varying concentrations of a commercial soil conditioner upon orange and lemon seedlings’ root growth, nutrient uptake, and overall citrus seedling physiology. Fundamentally, this research is a preliminary undertaking to better understand the strong and sometimes variable interactions between soil and plant physiology. In general, the statistical analysis confirmed that different levels of nutrients affected citrus seedling physiological indices. It is important to note that there were also different physiological responses based on genotype (orange vs. lemon seedling) and on product concentrations. 

In recent years, Florida growers have employed a plethora of strategies to increase citrus productivity. The most common of these strategies is increased soil and foliar fertilizer applications [27]. In addition, some growers apply soil conditioners, such as oGrowing© (SC), that can act as root growth enhancers. This product is applied in liquid form within the dripline of citrus seedlings. SC is comprised of dormant oceanic diatoms that, when applied to soil, assist the seedlings’ uptake of nutrients [25]. According to the chemical analysis of the product, SC also consists of 5% potassium (as reported in Materials and Methods).

The presence of oceanic diatoms in the product make it rich in silicon [28]. Although silicon (Si) is the second most abundant element on earth, its role in plant biology has not yet been fully understood [29,30]. Recent evidence suggests that Si is beneficial for the growth of higher plants, and several studies using Si have been conducted on citrus under salinity conditions. Results from these studies showed increases in photosynthesis and growth when plants were exposed to silicon [31,32]. Similar to citrus, other species have also shown positive responses to silicon. In particular, silicon nanoparticles were shown to boost the growth, total protein content and photosynthesis of lupin and wheat seedlings; results suggesting that Si was responsible for a change in the structure of the chlorophyll molecular configuration [33].

In addition to the Si provided by the SC, an increased availability of nutrients (N, P and K) is also responsible for changes in plant biomass. As reported by Boaretto et al. [34], when different citrus genotypes are exposed to the same fertilizer concentrations they respond in different ways. Their study reported that the dry mass measurements obtained from lemons was greater than that of oranges, when exposed to different concentrations of N. The same study also reported that oranges were less efficient in their use of N than lemons. Similarly, our study reported that orange seedlings increased in DW when exposed to lower concentrations of TF and SC, while lemon seedlings decreased in DW when exposed to lower concentrations of the TF. The failure to see a real impact of SC on lemon growth may be due in part to the short duration of the experiment; a 120-day period may be an insufficient length of time for all citrus genotypes to respond to treatments. It is characteristic of woody plants like lemon and orange to require longer research trials in comparison to faster growing (usually soft-stemmed) plants like vegetables and annuals. Furthermore, this confirms that the optimum amount of fertilizers to apply should be based on the genotype (orange vs. lemon) and not on the species [35]. This is especially important because both nutrient surpluses and deficiencies can affect overall plant performance, particularly orange and lemons which have different nutrient requirements [6]. 

When SC was applied in combination with TF, there was an increase in chlorophyll content and stomatal conductance in both orange and lemon genotypes. These gains were possibly caused by the increased availability of Si (as previously described), but also by the increased quantity of P in its available form [36,37]. It is important to note that, for our greenhouse experiment, we used Florida sandy soils collected from a citrus grove in order to mimic a natural environment (see Table 2); soils which are well known for the lack of available N and P for root uptake [38,39]. Phosphorus is needed for photosynthesis, synthesis, breakdown of carbohydrates, and the transfer of energy within the plant [40] and P losses due to surface runoff from Florida citrus groves have been a public concern since the early 1950s. In a study conducted using soils similar to those of our experiment, Yu et al. [39] indicated a clear loss of P as a result of surface runoff. However, soil data from a study on bearing Florida citrus orchards determined that many surface soils have, to a limited extent, the ability to retain applied P [38]. In addition, previous findings by Graham, Duncan and Eissenstat [35] demonstrated that P content in the soil is a key component for mycorrhization of citrus roots. Although a thorough study on mycorrhizae was not conducted in this particular experiment, it is well known that the presence of mycorrhizae aids nutrient uptake [41]. Therefore, the increased amount of available P taken up by roots may well be reflected in the elevated P levels of orange and lemon seedling stems that were treated with combined SC and TF. 

Generally, an increased availability of nutrients (N, P and K) is responsible for changes in physiological parameters [42,43,44]. Although changes can differ between genotypes, an increased level of chlorophyll *a*, *b* and total chlorophyll, and higher stomatal conductance was reported in both orange and lemon seedlings treated with combinations of soil conditioner and TF. Similar discoveries were found in Nemec and Vu [45], which indicated that citrus seedlings had increased photosynthetic activity when exposed to higher P concentrations. Bloomfield et al. [46] reported that tropical and subtropical soils are often characterized by low phosphorus availability, and tropical and subtropical seedlings typically exhibit lower rates of photosynthesis compared with plants growing in a different climate. In this specific regard, the increase in nutrient availability can be responsible for the recorded changes in chlorophyll content and stomatal conductance. 

In conclusion, the overall changes in plant physiology and plant growth can be directly connected with the increased availability of N, P and K, in conjunction with the silicon-rich SC. 

It is important to mention that this was the first study conducted on the effects of such soil conditioner on lemon and orange seedling physiological performance. With the continuing challenges that Florida’s citrus industry is facing, it is essential that new product claims be tested. Our results show that SC has a potential positive impact upon both chlorophyll levels and stomatal conductance values. However, based on dry weight growth data, we can conclude that the SC was effective for orange seedlings at 50% TF and 0.5% SC. Based on this short 120-day evaluation, the SC achieved positive growth promotion for orange (50% TF) but not for lemon seedlings. Although these results are promising, a field trial should be conducted in a commercial citrus grove to observe the effects of SC on older trees.

## 4. Materials and Methods

### 4.1. Plant Material and Treatments

‘Bearss’ lemon (*Citrus limon* (L.) Osbeck) and ‘Valencia’ sweet orange (*Citrus × sinensis* (L.) Osbeck) seedlings grafted on sour orange (*Citrus × aurantium* L.) rootstocks were employed. Sour orange seedlings were planted in March and budded on May. Before the start of the experiment, 13-2–13 fertilizer was rotated weekly with 20-10-20 fertilizer. This was applied through a drip system. Seedlings were maintained in a citrus nursery greenhouse (Murphy Citrus Nursery, Perry, FL) until they were 8 months old. At that time, seedlings were transported to the University of Florida, Institute of Agriculture and Life Sciences (UF/IFAS), Indian River Research and Education Center (IRREC) located in Fort Pierce, Florida (Latitude 27.426034, Longitude -80.408452). 

Five different treatments were designed to test the effects of varying TF (15-9-12) fertilizer concentrations in combination with a commercial soil conditioner (SC): 150 g/kg dry soil TF (Control), 150 g/kg dry soil TF + 1% SC, 150 g/kg dry soil TF + 0.5% SC, 75 g/kg dry soil TF + 1% SC and 75 g/kg dry soil TF + 0.5% SC. Five repetitions were used for each treatment, resulting in the testing of 25 lemons and 25 sweet orange seedlings. Information about mineral content of the two products can be found in Table 1.

Once received from the nursery, the lemon and orange seedlings were planted in Florida Flatwoods sandy soil typical of the Indian River region (Table 2). The soil was collected from the UF/IFAS IRREC experimental field. Before transplanting, seedlings were first rinsed and cleaned to remove nursery soil and then re-potted in black plastic nursery pots (27 cm wide × 31 cm tall). The pots were then numbered and labeled for each treatment group. The SC product was applied twice throughout the course of the experiment: the first application was administered at the beginning of the experiment (July 27), with a second application following two months later (September 27). 

### 4.2. Treatments and Cultivation

The plants were watered three times per day, five minutes each irrigation period. The irrigation flow was 32 ml per minute. Plants were watered automatically using the Hunter NODE (Hunter Industries, San Marco, CA, USA) irrigation control to set up irrigation time and frequency. Weed control was performed by hand every week, and soil in the top of pots were mixed to avoid the growth of algae. Miticide/Insecticide (Bifenthrin) was sprayed twice during the experiment. 

### 4.3. Vegetative Measurements

Height, diameter and stomatal conductance were measured every 15 days: on day 0 (D0), day 15 (D15), day 30 (D30), day 45 (D45), day 60 (D60), day 75 (D75), day 90 (D90), day 105 (D105), and day 120 (D120). These measurements were made on all experimental plants at each testing period. Height was measured from the soil surface to the top of the highest leaf. Stem diameter was measured using a caliper (Neiko 6” Stainless Steel Digital Caliper, Neiko Tools USA, China). Stomatal conductance was measured at 12.00 PM (midday, noon) using the (Decagon Devices model SC-1, Pullman, WA, USA), with measurements taken from one leaf located in the mid-section of each plant. 

At the end of the experiment, the plants were removed from their pots. The root systems were rinsed off with deionized (DI) water. Each plant was then divided into three different parts: root, stem and leaf. All the parts were weighed separately on an analytical scale (Sartorius AG, Göttingen, Germany) to obtain fresh weights. The samples were then placed in separate labeled brown paper bags. All the samples inside the brown paper bags were put in a 70° C drying oven for one week. Dry weights were then collected.

### 4.4. Mineral Analysis

The dry samples were analyzed for nutrient concentrations [47,48]. Nitrogen (N) concentration (%) was determined using a NA2500 carbon (C)/N Analyzer (Thermoquest CE Instruments; ThermoQuest Corporation, Thermo Fisher Scientific Inc., Waltham, MA, USA). Tissue phosphorus (P) and potassium (K) concentration was determined using a dry ash combustion digestion method [49]. A 1.5 g sample of dried plant material was weighed and dry-ashed at 500 °C for 16 h. The ash was equilibrated with 15 ml of 0.5 M hydrochloric acid (HCl) at room temperature for 0.5 h. The solution was decanted into 15 ml plastic conical tubes and placed in a refrigerator at 4 °C until analyses by inductively coupled plasma atomic emission spectrometry (ICP-AES) could be performed [50].

### 4.5. Chlorophyll Contents

Circular leaf samples were cut from leaves of each plant harvested. The cut lamina was then placed in 25 mL of dimethylformamide (DMF) and kept at 4° C in the dark. After 48 hours, the samples were tested in a UV-visible spectrophotometer (Thermoscientific Genesys 50, Hampton, NH, USA) in quartz cuvettes at 664 and 647 nm. The resulting readings were then put into the following formulae [51,52]:

Chl *a* = 12.70 × A_664_ − 2.79 × A_647_ (1)

Chl *b* = 20.70 × A_647_ − 4.62 × A_664_ (2)

Total Chl = 17.90 × A_647_ + 8.08 × A_664_ (3)

### 4.6. Experimental Design and Statistical Analysis

The experiment was placed inside a greenhouse and was set up using a completely randomized experimental design (*n* = 5). One-way ANOVA was performed and mean separation between treatments was obtained using Tukey’s test (*p* ≤ 0.05). 

## Figures and Tables

**Table 1:** Information about labels and mineral content (N-P-K) of the two products used in this study.

Products	Traditional Fertilizer (TF; Osmocote® Plus)	Soil Conditioner (SC; oGrowing™)
**Total Nitrogen (N) %** *Ammoniacal Nitrogen %* *Nitrate Nitrogen*	**15.00** *8.40* *6.60*	**0.19** *0.07* *0.12*
**Available Phosphorus P_2_O_5_**	**9.00**	**n/a**
**Soluble Potassium K_2_O**	**12.00**	**5.21**

**Table 2:** Information about the mineral content of the sandy soils used in this study.

Element	Content (mg/Kg of Dry Soil)
Nitrogen (N)	3.35
Phosphorus (P)	13.5
Potassium (K)	30.33
